# Exposure of Free-Ranging Wild Animals to Zoonotic *Leptospira interrogans* Sensu Stricto in Slovenia

**DOI:** 10.3390/ani11092722

**Published:** 2021-09-17

**Authors:** Diana Žele-Vengušt, Renata Lindtner-Knific, Nina Mlakar-Hrženjak, Klemen Jerina, Gorazd Vengušt

**Affiliations:** 1 Institute of Pathology, Wild Animals, Fish and Bees, Veterinary Faculty, University of Ljubljana, Gerbičeva 60, 1000 Ljubljana, Slovenia; diana.zelevengust@vf.uni-lj.si; 2 Institute of Poultry, Birds, Small Mammals and Reptiles, Veterinary Faculty, University of Ljubljana, Gerbičeva 60, 1000 Ljubljana, Slovenia; renata.lindtnerknific@vf.uni-lj.si (R.L.-K.); nina.mlakarhrzenjak@vf.uni-lj.si (N.M.-H.); 3 Department of Forestry and Renewable Forest Resources, Biotechnical Faculty, Večna pot 83, 1000 Ljubljana, Slovenia; klemen.jerina@bf.uni-lj.si

**Keywords:** wildlife, *Leptospira interrogans*, microscopic agglutination test, serology, Slovenia

## Abstract

**Simple Summary:**

Wildlife can serve as a reservoir for highly contagious and deadly diseases, many of which are infectious to domestic animals and/or humans. Wildlife pathogen and disease surveillance is, thus, an essential tool that can provide valuable information on population health status and protect human health. Blood samples from 244 wild animals and 5 from carcasses were tested for specific antibodies against *Leptospira* serovars in Slovenia between 2019 and 2020 using the microscopic agglutination test. The results confirm that various wildlife species were exposed to *Leptospira interrogans* and may be used as a sentinel for leptospirosis, which is considered a significant health threat to other wildlife species and to humans.

**Abstract:**

A total of 249 serum samples from 13 wild animal species namely fallow deer (*Dama dama*, *n* = 1), roe deer (*Capreolus capreolus*, *n* = 80), red deer (*Cervus elaphus*, *n* = 22), chamois (*Rupicapra rupicapra*, *n* = 21), mouflon (*Ovis musimon, n* = 4), brown hare (*Lepus europaeus*, *n* = 2), nutria (*Myocastor coypus*, *n* = 1), red fox (*Vulpes vulpes*, *n* = 97), stone marten (*Martes foina*, *n* = 12), European badger (*Meles meles*, *n* = 2), golden jackal (*Canis aureus*, *n* = 2) Eurasian lynx (*Lynx lynx*, *n* = 2) and grey wolf (*Canis lupus*, *n* = 3) were analysed for the presence of antibodies against *Leptospira interrogans* sensu stricto. Serum samples were examined via the microscopic agglutination test for the presence of specific antibodies against *Leptospira* serovars Icterohaemorrhagiae, Bratislava, Pomona, Grippotyphosa, Hardjo, Sejroe, Australis, Autumnalis, Canicola, Saxkoebing and Tarassovi. Antibodies to at least one of the pathogenic serovars were detected in 77 (30.9%; CI = 25–37%) sera. The proportion of positive samples varied intraspecifically and was the biggest in large carnivores (lynx, wolf and jackal; 86%), followed by mezzo predators: stone marten (67%) and red fox (34%), and large herbivores: red deer (32%), roe deer (25%), alpine chamois (10%) and mouflon (0%). Out of the 77 positive samples, 42 samples (53.8%) had positive titres against a single serovar, while 35 (45.4%) samples had positive titres against two or more serovars. The most frequently detected antibodies were those against the serovar Icterohaemorrhagiae. The present study confirmed the presence of multiple pathogenic serovars in wildlife throughout Slovenia. It can be concluded that wild animals are reservoirs for at least some of the leptospiral serovars and are a potential source of leptospirosis for other wild and domestic animals, as well as for humans.

## 1. Introduction

In recent decades, international attention on wildlife diseases, including surveillance and monitoring programmes, has increased [1,2]. Wildlife diseases occur in numerous forms in a wide range of species and populations around the globe. Leptospirosis is a zoonosis of global importance, affecting many species of wild and domestic animals, as well as humans [1,3]. *Leptospira* spp. are also considered as small mammal-associated zoonotic pathogens causing diseases with potentially similar symptoms in humans [4]. It is considered as one of the most important re-emerging health threats to humans by the World Organisation for Animal Health [5,6]. Various pathogenic serovars of *Leptospira* have been serologically classified into 22 serogroups and over 300 serovars based on the microscopic agglutination test (MAT) or the cross-agglutination absorption test (CAAT), respectively [7], with each serovar tending to be maintained by a host group and capable of causing the disease [8,9,10]. Small rodents are the usual reservoirs of leptospires in natural herds [11,12,13]. Data from the Netherlands show that insectivores and rodents could serve as indicators of environmental contamination and/or wildlife contamination with *Leptospira* spp. [14]. Studies worldwide indicate that various wild ruminants [15,16,17,18], lagomorphs [15,18] and carnivores [15,19,20,21] are also potential sources of leptospires. Wildlife species are generally considered to be important epidemiological vectors, mainly because of their frequent reactivity to *Leptospira* serovars native to their habitat [16]. These reservoirs are thought to act as a source of infection for humans and domestic animals, who can then become a source of infection for other animals and humans [6,11]. However, data on the epidemiology of *Leptospira* infections in wildlife and the public health significance of wildlife species worldwide are lacking [20,22].

The presence of antibodies in wild animals may indicate previous or current infection, which may have occurred either by direct contact with the contaminated urine of another animal or by the consumption of infected prey [23]. Several domestic and wild animals become infected and, thus, become kidney carriers, excreting the pathogen through their urine [23] and via parent–offspring transmission [24]. Wildlife are reservoir hosts for leptospires and often show no clinical signs of disease; however, these reservoirs can serve as a source of infection for humans and domestic animals, who can then become a source of infection for other animals and humans [5,6,25]. In addition, there are few data on Leptospira antibodies and infections in wildlife, although transmission to livestock and humans often originates from or is maintained by wildlife [25].

Humans may be exposed to *Leptospira* infections directly through contact with infected material or indirectly through the contaminated environment [26,27]. Studies have shown that peak incidence of disease occurred after periods of excessive rainfall and flooding [28]. Infections with *Leptospira* spp. can affect not only people in exposed professions (i.e., veterinarians, trappers, abattoir workers, farm workers, hunters, animal shelter workers and scientists and technicians involved with animals in laboratories or in the field) but also people who work with marine mammals, fishmen, researchers, wildlife rehabilitators, animal trainers and zoological park workers [3]. A study conducted in Austria [29] reported that hunters in particular are exposed to zoonotic agents, including leptospires, probably through the direct contact of abraded skin or mucous membranes with the tissues, blood or urine of infected animals [30,31]. In some cases, leptospirosis can present as a severe disease in both animals and humans and can lead to death [11]. Human infections by *Leptospira* spp. and orthohantaviruses are almost indistinguishable in their clinical presentation [32] and can often be confused with each other [4]. In addition, coinfections with orthohantaviruses are frequently observed at sites where the prevalence of *Leptospira* spp. in small mammals exceeded 35% [4]. Therefore, long-term active surveillance and studies of wildlife reservoirs will help to understand the role of wildlife as a reservoir and as a source of leptospires and other pathogens to humans [13]. According to Podgoršek [33], the epidemiological picture in Slovenia is comparable to that in Europe. Up to 30 cases of *Leptospira* infection with the predominant serovars Grippotyphosa and Icterohaemorrhagiae and the species *L. kirschneri* and *L. interrogans* sensu stricto are reported annually [33,34]. In Slovenia, the risk of contracting leptospirosis is associated with occupational and recreational exposure [34].

MAT is considered the gold standard for sero-diagnosis of leptospirosis because of its unsurpassed diagnostic specificity [35]; however it is not sufficiently sensitive for the diagnosis of the acute phase of the disease [36]. It would be an important tool for epidemiological purposes, such as identifying infecting serovars [36]. Antigens can be detected by histological, histochemical or immunostaining techniques. Unfortunately, none of these tests are currently suitable for routine laboratory use because of technical limitations and low sensitivity [37]. Isolation of *Leptospira* from the clinical specimen is difficult because leptospires are fastidious, slow growing and require special growth media, and it is time consuming and laborious [38]. Therefore, PCR assay is very useful as a contemporary method for diagnosis in the acute phase of leptospirosis [39].

To date, studies in Slovenia have confirmed the presence of specific antibodies against 11 pathogenic *Leptospira* serovars in wild boar [40], but there is currently no information on the seroprevalence and distribution of leptospirosis in other wildlife species in Slovenia. The aim of this study was to investigate the seroprevalence of pathogenic *L. interrogans* serovars in different wild species in Slovenia.

## 2. Materials and Methods

### 2.1. Samples

**Figure 1:**
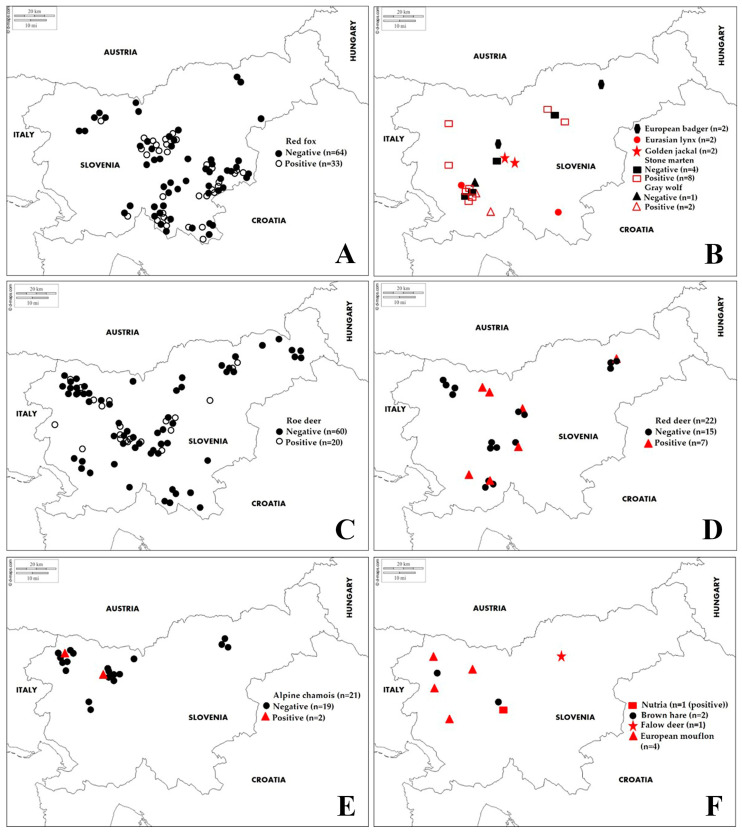
Geographical location of *Leptospira interrogans* antibody-negative and -positive samples of different wildlife species (**A**)—red fox; (**B**)—European badger, Eurasian lynx, golden jackal, stone marten, grey wolf; (**C**)—roe deer; (**D**)—red deer; (**E**)—chamois; (**F**)—nutria, brown hare, fallow deer, European mouflon) detected by MAT in Slovenia from 2019 to 2020.

During the 2019 and 2020 hunting season (May to December), blood samples were collected nationwide from a total of 244 apparently healthy, free-ranging wild animals, and 5 clotted blood samples were collected from carcasses (Table 1 and Figure 1). Licensed game wardens and hunters were invited to submit samples from animals shot during the regular annual cull or from animals found dead in nature. Prior to sample collection, hunters were instructed on procedures and were provided with field sample kits. Immediately after animal death, blood samples were collected from the jugular vein or heart. As a part of the national passive health surveillance of wildlife in Slovenia, carcasses of wild large predators (Eurasian lynx and grey wolf) found dead in their habitats were sent for necropsy to the Veterinary Faculty, University of Ljubljana. Clotted blood samples were taken from the heart. The authors declare that no animals were killed for the purpose of this study and that all procedures contributing to this work met the ethical standards of the relevant national and European regulations on the care and use of animals (Directive 2010/63/EC).

### 2.2. Laboratory Methods

After field collection, blood samples were transported to the Veterinary Faculty, University of Ljubljana, within 24 h. Many of the collected samples were haemolysed and were, therefore, rejected at the pre-analysis stage (*n* = 34). Fresh blood samples and clotted blood samples were centrifuged at 4000 rpm for 15 min (LC 320) to obtain the sera. Sera were transferred with serum pipettes into sterile Eppendorf tubes and stored at −20 °C before being tested for the presence of specific antibodies against pathogenic serovars of *Leptospira interrogans* sensu stricto using the MAT.

Live cultures of different serovars were used as antigens: Grippotyphosa, strain Moskva V; Sejroe, strain Mallerdorf 84; Pomona, strain Pomona; Tarassovi, strain Mitis Johnson; Copenhageni (serological group: Icterohaemorrhagiae), strain Wijnberg; Canicola, strain Hond Utrecht IV; Australis, strain Ballico; Autumnalis, strain Akyami A; Bataviae, strain Van Tienen; Saxkoebing, strain MUS 24; Bratislava, strain Jež Bratislava; and Hardjo, strain Hardjo Bovis. The MAT was performed according to the accredited method in accordance with the protocol standard operating procedure (SOP 120) in the laboratory for leptospirosis at the Veterinary Faculty in Ljubljana and was carried out in two phases. In the first phase (pretest), the presence of specific antibodies for the serovars used in the test was determined, while in the second phase, a twofold titration of positive sera, starting with the dilution 1:50, was performed. Phosphate buffer (PBS; Dulbecco’s phosphate-buffered saline, Sigma-Aldrich, Burlington, MA, USA) was used for serum dilutions. Results were read using a darkfield microscope with a magnification of 160, and the endpoint was estimated as 50% agglutination or the lysis of leptospires in the microscopic field. Samples that had titres of ≥50 against one or more serovars were considered positive.

### 2.3. Statistical Analyses

Estimates and confidence intervals (CIs, for *p* = 0.05) of the estimated proportions of individuals exposed to *L. interrogans* for each of the species studied were calculated, taking into account the binomial distribution of the exposure outcome (yes/no). Confidence intervals were estimated only for the species with adequate sample size. Differences in the extent of exposure to *L. interrogans* among the animal species studied were also investigated. These differences were evaluated with chi-square tests of homogeneity, first for all species together, and in the second phase, for all combinations of pairs of species. Test of homogeneity provides reliable results if the theoretical frequency in each cross-section of the levels of tested variables is larger than 1. To reach this condition, species with very small sample size (*n* < 4) were either excluded from analysis (fallow deer, badger, hare and nutria) or, in the case of species with similar biology and thus expected similar prevalence for examined disease, data from several species were pulled together in a wider group, e.g., species Eurasian lynx, grey wolf and golden jackal were joined in new group named “large carnivores”.

## 3. Results

Examination of 249 blood sera from wild animals revealed antibodies to at least one of the pathogenic serovars in 77 sera (30.9%; CI 25.2–36.7%) (Table 2 and Figure 1). Of the 77 positive samples, 42 samples (53.8%) had positive titres against a single serovar, while 35 (45.4%) samples had positive titres against two or more serovars. Of all positive reactions, the highest antibody seroprevalence was found for serovar Australis in red fox and stone marten; serovar Icterohaemorrhagiae in golden jackal, grey wolf and roe deer; serovar Pomona in red deer; serovars Icterohaemorrhagiae and Sejroe in Eurasian lynx; serovars Icterohaemorrhagiae and Tarassovi in chamois; and serovar Bratislava in nutria. Serovar Icterohaemorrhagiae showed the highest antibody titre, 1:6400 in red fox (Table 3). No antibodies were detected in fallow deer, European badger, European mouflon or brown hare.

A test of homogeneity shows that there are differences in the proportion of positive cases between different animal species (Pearson chi-square = 25.6; df = 7; *p* = 0.00059). Overall, the largest proportion of positive cases was detected in the large carnivores group (86%; CI = 60–100%; *n* = 7), followed by two medium-sized predator species: stone marten (67%; CI = 40–93%; *n* = 12) and red fox (34% CI = 25–43%; *n* = 97), and in declining order the following four species of large herbivores: roe deer (25%; CI = 16–34%; *n* = 80), red deer (32%; CI = 12–51%; *n* = 22), alpine chamois (10%; CI = 0–22%; *n* = 21) and as the last European mouflon with no detected positive cases (0%; *n* = 4). Other species had too low a sample size for statistical analysis. The single analysed sample of nutria was positive and the sample of fallow deer was negative for the presence of specific antibodies against *Leptospira* serovars; moreover, both results of both tested samples of brown hare and European badgers were negative.

Paired comparisons between species proved differences between large carnivores and different species of large herbivores, between stone marten and species of large herbivores, and between red fox and alpine chamois (*p* < 0.05), but it is noteworthy that outcomes of the formal statistical test depend on sample size and are prone to type I error in cases of small samples (Table 4).

## 4. Discussion

Wildlife and domestic animals play an important role as the reservoir for particular *Leptospira* serovars. Environmental characteristics, topography, meteorology, human presence and species interactions can influence the occurrence and density of *Leptospira* species [41]. In wild animals, specific climatic, edaphic and hydrological factors also determine the incidence of leptospirosis in different habitats [42]. Differences in prevalence and serovars between studies in wildlife could be due to inconsistencies in cutoff titres, serovars tested, sampling site characteristics, climate or geographic location and timing of the year of the study [43].

In this study, blood samples were taken at random from animals shot during the regular annual cull or found dead in the wild without any knowledge of possible infection or its duration. Determination of antibody titre by MAT has been used as a tool for leptospirosis diagnosis. Different diagnostic tests that can be used to detect leptospirosis have advantages and disadvantages, and laboratory diagnosis of leptospirosis is challenging. A positive culture of biological samples (blood, urine, tissue) is the definitive proof of infection, but culturing leptospires is laborious and fastidious. The bacterium requires special growth media, and incubation can last for months [44]. Histopathological examination of the kidneys is not indicated to replace the serological diagnosis of leptospirosis, and may be used only as a complementary examination [45]. In the early stages of the disease, the only sensitive and specific test is PCR. Its limitation is that it does not detect DNA in the blood during the first 5–10 days after the onset of the disease and until the 15th day [37]. MAT is the most commonly used serological test in the diagnosis of leptospirosis, despite being a technically demanding and laborious procedure [46]. MAT can be positive from the 10th to 12th day after disease onset and can detect both class M and class G antibodies [37]. MAT has a sensitivity of 41% in the first week, 82% in the second to fourth week and 96% after the fourth week of illness [47]. MAT is considered the gold standard for serodiagnosis of leptospirosis due to its unsurpassed diagnostic specificity [35], but it is not sufficiently sensitive for diagnosis of the acute phase of the disease [36]. Another limitation of serology is that it cannot distinguish between current, recent or previous infections [37]. In the present study, paired samples to confirm acute or convalescent infection were not available.

The analyses performed in the present study showed that most of the carnivores studied (with the exception of the European badger) and the most abundant wild ruminants in Slovenia were frequently exposed to *L. interrogans*. The significant percentage of seropositive red fox, stone marten, grey wolf and other carnivores tested may indicate that those species are reliable sentinels for epidemiological monitoring in Slovene forest habitats, which can also explain positive titres to *L. interrogans* in roe deer and red deer sharing the same biotope. Infection by multiple serogroups was confirmed, suggesting that multiple epidemiological cycles exist in the Slovenian region. The results of our study confirmed antibodies against 10 pathogenic *Leptospira* serovars in carnivores, 8 in wild ruminants and 1 in nutria. The observed seroprevalence of leptospiral antibodies in the tested wildlife species could not be extrapolated to the whole population level in Slovenia due to the statistically insufficient number of samples but could be a good indicator of the importance of these wildlife species in leptospirosis transmission.

The red fox (*Vulpes vulpes*) is widely distributed in European countries [48] and is the most widespread mesopredator in Slovenia. Since 2013, the hunting bag of red fox increased from 10.400 to 15.715 in 2019 [49,50]. Several serological studies using MAT have shown that red foxes are frequently exposed to different serovars of *Leptospira* spp. [19,21,51,52,53]. The results of our study confirm that interactions between different *Leptospira* serovars are also common in the Slovenian red fox population. The seroprevalence of antibodies to leptospiral serovars (34%) found in this study was lower than the seroprevalence reported in red foxes from Spain (47.1%) [19] but higher than that in other European countries, such as Poland (26.3%) [21], Croatia (31.25%) [33], Norway (9.9%) [51] and Germany (1.9%) [52], all using MAT. The different results from the different countries are difficult to explain because research on the seroprevalence of *Leptospira* spp. in foxes requires extensive ecological knowledge of fox population dynamics and must include considerations of juvenile fall migrations, home range, population density, litter size, yearly accession, mortality rate and hunting pressure [53]. It is also very important to consider epidemiological data on leptospiral archaic foci, reservoirs, maintenance hosts and serovar distribution [42]. Antibodies against serovar Australis (51.5%) were detected most frequently, followed by those against serovar Bratislava (48.5%) and Icterohaemorrhagiae (33.3%). Antibodies against serovar Australis were also the most frequently detected antibodies in red foxes in Croatia [54]. According to data from studies in other European countries, the most common serovar in red foxes is Icterohaemorrhagiae [19,51], while the Bratislava serovar is less common in Europe. The exposure of foxes to this serovar is not surprising because rodents, an important food source for foxes, are probably the most important host for a variety of *Leptospira* serovars in rural and urban environments [55,56].

Among all European members of the marten family, the stone marten is the only species whose population is increasing, and it is one of the most widespread mustelids in the Eurasian region [57]. The current population size is unknown. The results of our study confirmed the presence of specific antibodies against various serovars of *Leptospira* in the stone marten population. *Leptospira* antibodies were found in eight animals, a seroprevalence of 66.6% (8/12). The high seroprevalence in stone martens in Slovenia is comparable to that in Croatia (4/7; 62.50%) [42], while in Spain and France, all samples (*n* = 8) were negative against *L. interrogans* serovars using MAT [18,58]. In Slovenia, antibodies against serovar Australis (66.66%) were most frequently detected in stone martens, followed by those against serovar Bratislava (50%) and Icterohaemorrhagiae (25%).

The golden jackal (*Canis aureus*) is one of the most widespread canid species [59] and has also established territories in Slovenia [60]. A rough estimate of the population size in Slovenia is about 1000 individuals [61]. The results of our study confirmed the presence of specific antibodies against different serovars of *Leptospira* in the golden jackal population. *Leptospira* antibodies were found in two animals with a seroprevalence of 100% (2/2). The study data indicated that golden jackals could transmit different *Leptospira* serovars. They showed titres against Icterohaemorrhagiae, Pomona, Hardjo, Sejroe and Saxkoebing serovars. The seroprevalence of antibodies against leptospiral serovars found in this study was comparable to the seroprevalence reported in golden jackals from Ukraine (100%; 9/9). Both golden jackals tested had titres against five serovars [62].

The grey wolf *(Canis lupus*) is the largest wild member of the dog family (*Canidae*). Its population in Slovenia is increasing and includes over 100 individuals [63]. *Leptospira* antibodies were found in two animals with a seroprevalence of 66.6% (2/3). Serological reactions for the Grippotyphosa, Pomona and Icterohaemorrhagiae serogroups were detected in a grey wolf in Italy [64]. Few studies have been conducted in the United States of America, where seroprevalence in grey wolves ranged from 1% to 11% [65,66], and the most commonly detected serovar was Grippotyphosa.

The Eurasian lynx (*Lynx lynx*) is the third-largest predator in Europe after the brown bear and grey wolf. The population in Slovenia is estimated at only about 15 individuals [67]. *Leptospira* antibodies were found in two animals with a seroprevalence of 100% (2/2). The study data in Eurasian lynx showed low titres of antibodies against serovars Icterohaemorrhagiae and Sejroe. Seroprevalence in Iberian lynx in Spain [19] and Quebec in wild lynx (*Lynx canadensis*) was 32% (7/22) and 1% (1/97), respectively. In Spain, the most frequently detected serovars were Icterohaemorrhagiae, and in Quebec, in one case, Pomona and Bratislava. According to Labelle et al. [68], the low seroprevalence of antibodies to *L. interrogans* in lynx is unexpected because rodents, one of the main food sources for these animals, are known reservoirs of *L. interrogans*. Throughout Europe and in Slovenia, the diet of lynx usually consists of European roe deer, which is clearly the preferred prey of Eurasian lynx [69]. In this study, the seroprevalence of antibodies to *L. interrogans* in roe deer was 25%. We believe that roe deer may also serve as a source of leptospirosis for lynx.

The European roe deer (*Capreolus capreolus*) is the most common and widespread deer species in Europe [70]. The rough estimate of roe deer population size in Slovenia is about 110,000 individuals [71]. In recent decades, the population size and, at the same time, the hunting bag of roe deer have greatly increased in most parts of Europe [72]. Roe deer is one of the most important game species and a crucial prey of large carnivores in Europe [70,73]. In roe deer, a seroprevalence of 25% (20/80) was observed. Animals showed titres against five serovars. The majority of the positive samples had positive titres against a single serovar. Antibodies against serovar Icterohaemorrhagiae (65%) were most frequently detected. The seroprevalence of antibodies against leptospiral serovars found in this study was considerably higher than that in Croatia (6.0%) [42] or in Poland, which showed an overall seroprevalence in deer (roe deer, red deer and fallow deer) of 4.8% [74], or in Germany (2%) [75]. No positive serological reactions were found in roe deer (*n* = 66) in Italy [17].

The red deer (*Cervus elaphus*) is the second most abundant deer species in almost all of Europe [76]. The rough estimate of the population size is 10,000–14,000 animals [77]. A seroprevalence of 31.8% (7/22) was found in red deer. The animals showed titres against seven serovars. Antibodies against serovar Pomona (42.8%) were detected most frequently. The seroprevalence of antibodies against leptospiral serovars found in this study was significantly higher than that in Italy (6.33%) [17] or in Poland, which showed a total seroprevalence in deer (roe deer, red deer and fallow deer) of 4.8% [74], or in Croatia (19.02%) [42].

Chamois (*Rupicapra rupicapra*) is a habitat-specialised ungulate inhabiting “continental archipelagos” with fragmented rocky habitats, often restricted to high altitudes [78]. The estimated number of chamois in Slovenia is 10,000 [77]. *Leptospira* antibodies were found in two animals, a seroprevalence of 9.52% (2/21). Study data revealed that chamois had antibodies against serovars Icterohaemorrhagiae and Tarassovi. In Italy, no positive serological reactions for *Leptospira* serovars were found in chamois (*n* = 138) [17]. To our knowledge, our study is the first to report positive samples in chamois for *Leptospira* antibodies. It is, however, noteworthy that the proportion of positive samples in chamois was among the lowest between all analysed species in Slovenia being followed only by mouflon (0/4).

The coypu, also known as the nutria (*Myocastor coypus*), is a semiaquatic rodent and significant carrier of pathogenic *Leptospira* in Europe [79]. The current population size is unknown. We tested one animal and detected antibodies against serovar Bratislava. Several researchers in Europe have presented information on antibodies to leptospiral serovars in nutria, ranging from 11.5% in Italy [80] to 76% in France [81] with the predominance of the Icterohaemorrhagiae serogroup. These results are consistent with the idea that nutria should be considered a risk factor for leptospirosis in humans and domestic animals and should be taken into account by public health decision makers, especially with regard to prevention and population control [79].

Unlike clinical disease seen in canines and humans, the health impact of leptospirosis in wildlife is unclear [43]. Necropsy of fresh carcasses of animals killed in traffic accidents to look for renal lesions [19] and collection of tissue for immunohistochemistry would help in confirming the disease [82].

The risk of contracting leptospirosis is associated with occupational and recreational hazards [34]. Strategies for leptospirosis prevention are, therefore, based on education about the epidemiology and transmission mechanisms of leptospirosis [83]. Education of occupationally exposed workers about contact with contaminated water or infected animals is particularly important. Personal protective measures should also be taken for workers in high-risk occupations. The risk of infection can be reduced by increasing awareness of the routes of infection, avoiding contact with high risk water sources and using of prophylaxis during high-risk activities [84]. Increased efforts should be made to identify and treat infected animals at an early stage and to raise awareness of immunisation options for domestic and farm animals [83].

## 5. Conclusions

Our data confirm that large and medium-sized carnivores are frequently exposed to the pathogenic serovars of *L. interrogans* and may play the role of sentinel for leptospirosis. Data on *L. interrogans*-specific antibody-positive wild ruminants suggest that these species, although less infected, may still be a potential source of leptospirosis for humans, with the risk of infection particularly high for veterinarians, butchers, people working in forested areas and, frequently overlooked, hunting dogs. Due to the small number of samples tested, further investigation of the prevalence of infection in wild animals in Slovenia is needed to clarify the epidemiological significance of wild animals for leptospirosis transmission.

## Figures and Tables

**Table 1:** Samples from 249 free-ranging wild animals, harvested or found dead (*).

Species Common Name	Latin Name	No. of Animals
Alpine chamois	*Rupicapra rupicapra*	21
Brown hare	*Lepus europaeus*	2
European badger	*Meles meles*	2
European mouflon	*Ovis musimon*	4
Fallow deer	*Dama dama*	1
Golden jackal	*Canis aureus*	2
Nutria	*Myocastor coypus*	1
Red deer	*Cervus elaphus*	22
Red fox	*Vulpes vulpes*	97
Roe deer	*Capreolus capreolus*	80
Stone marten	*Martes foina*	12
* Eurasian lynx	*Lynx lynx*	2
* Grey wolf	*Canis lupus*	3
Total samples		249

**Table 2:** Number of serum samples (Total no.) collected from different wildlife species in Slovenia from 2019 to 2020, testing positive (No. pos.) to antibody against *Leptospira* serovars.

Common Name	Total No.	No. Pos.	Proportion of Positives (and CI)	Serovars								
Alpine chamois	21	2	10 (0–22)%	Ictero	Brat	Tar	-	-	-	-	-	-
Brown hare *	2	0	-	-	-	-	-	-	-	-	-	-
European badger *	2	0	-	-	-	-	-	-	-	-	-	-
European mouflon *	4	0	-	-	-	-	-	-	-	-	-	-
Fallow deer *	1	0	-	-	-	-	-	-	-	-	-	-
Red deer	22	7	32 (12–51)%	Ictero	Brat	Pom	Grip	Sejroe	Aut	Can	-	-
Roe deer	80	20	25 (16–34)%	Ictero	Brat	Pom	Grip	Sejroe	-	-	-	-
European badger *	2	0	-	-	-	-	-	-	-	-	-	-
Eurasian lynx	2	2	Large carnivores 86 (60–100)%	Ictero	Sejroe	-	-	-	-	-	-	-
Golden jackal	2	2		Ictero	Pom	Hardjo	Sejroe	Sax	-	-	-	-
Gray wolf	3	2		Ictero	Grip		-	-	-	-	-	-
Red fox	97	33	34 (25–43)%	Ictero	Brat	Pom	Grip	Sejroe	Aus	Aut	Sax	Can
Stone marten	12	8	67 (40–93)%	Ictero	Brat	Pom	Aus	Sax	-	-	-	-
	249	77	30.9 (25.2–36.7)%									

* The proportion and CI of positive samples are not presented due to unreliability resulting from small sample size. Abbreviations: Icterohaemorrhagiae (Ictero), Bratislava (Brat), Pomona (Pom), Grippotyphosa (Grip), Australis (Aus), Autumnalis (Aut), Canicola (Can), Saxkoebing (Sax) and Tarassovi (Tar).

**Table 3:** Serovars and antibody titres against *Leptospira* serovars in tested wild animals.

Common Name	Serovars	Titre					
		50	100	200	400	800	≥1600
Alpine chamois	Ictero	0	1	0	0	0	0
	Tarassovi	1	0	0	0	0	0
Eurasian lynx	Ictero	0	1	0	0	0	0
	Sejroe	1	0	0	0	0	0
Golden jackal	Ictero	0	1	1	0	0	0
	Pomona	0	0	0	1	0	0
	Hardjo	0	1	0	0	0	0
	Sejroe	0	0	0	0	1	0
	Saxkoebing	0	0	0	1	0	0
Gray wolf	Ictero	1	1	0	0	0	0
	Grippo	0	1	0	0	0	0
Nutria	Bratislava	0	1	0	0	0	0
Red fox	Ictero	3	2	3	0	1	2
	Bratislava	2	4	7	2	1	0
	Pomona	2	1	3	2	0	0
	Grippo	1	0	0	0	0	0
	Sejroe	2	4	2	1	0	0
	Australis	7	1	6	0	2	1
	Autumnalis	0	0	1	0	0	0
	Canicola	0	1	0	1	0	0
	Saxkoebing	0	2	3	1	0	0
Red deer	Ictero	0	1	0	0	0	0
	Bratislava	0	1	0	0	0	0
	Pomona	1	1	0	1	0	0
	Grippo	1	0	0	0	0	0
	Sejroe	1	0	0	0	0	0
	Autumnalis	0	1	0	0	0	0
	Canicola	1	1	0	0	0	0
	Bratislava	2	1	3	0	0	0
	Pomona	0	0	1	0	0	0
	Australis	2	1	1	1	2	1
	Saxkoebing	1	0	0	0	0	0
Roe deer	Ictero	8	4	1	0	0	0
	Bratislava	4	0	0	0	0	0
	Pomona	1	0	0	1	0	0
	Grippo	1	0	0	0	0	0
	Sejroe	1	0	0	0	0	0
Stone marten	Ictero	0	2	0	1	0	0

**Table 4:** Outcome of chi-square homogeneity tests of differences in proportion of detected antibodies for *L. interrogans* between pairs of species. Numbers in the table are *p*-values; significant differences are in bold. The proportion of positive cases is given in parentheses.

Species (Proportion of Positive Cases)	Mouflon (0%)	Alpine Chamois (10%)	Roe Deer (25%)	Red Deer (32%)	Red Fox (34%)	Stone Marten (67%)	Large Carnivores (86%)
Mouflon (0%)	1.000	0.526	0.255	0.199	0.158	**0.037**	**0.027**
Alpine chamois (10%)	0.526	1.000	0.129	0.080	**0.028**	**0.002**	**0.001**
Roe deer (25%)	0.255	0.129	1.000	0.522	0.194	**0.004**	**0.001**
Red deer (32%)	0.199	0.080	0.522	1.000	0.844	0.059	**0.019**
Red fox (34%)	0.158	**0.028**	0.194	0.844	1.000	0.030	**0.008**
Stone marten (67%)	**0.037**	**0.002**	**0.004**	0.059	**0.030**	1.000	0.376
Large carnivores (86%)	**0.027**	**0.001**	**0.001**	**0.019**	**0.008**	0.376	1.000

## Data Availability

The data presented in this study are available upon request from the corresponding author.
